# Identification of a TCR signature in peripheral blood derived CD4+ T cells, associated with chronic chikungunya disease, suggests a conducive, female-biased, background immune profile

**DOI:** 10.3389/fimmu.2026.1739100

**Published:** 2026-03-11

**Authors:** Koen Bartholomeeusen, Fabio Affaticati, Elisabeth Willems, Emilie Dhondt, Esther Bartholomeus, Alvino Maestri, Sowath Ly, Duong Veasna, Benson Ogunjimi, Pieter Meysman, Kris Laukens, Tineke Cantaert, Kevin K. Ariën

**Affiliations:** 1 Virology Unit, Department of Biomedical Sciences, Institute of Tropical Medicine, Antwerp, Belgium; 2 Antwerp Center for Translational Immunology and Virology (ACTIV), Vaccine and Infectious Disease Institute, University of Antwerp, Antwerp, Belgium; 3 Adrem Data Lab, Department of Mathematics and Computer Science, University of Antwerp, Antwerp, Belgium; 4 Biomedical Informatics Research Network Antwerp (biomina), University of Antwerp, Antwerp, Belgium; 5 Antwerp Unit for Data Analysis and Computation in Immunology and Sequencing (AUDACIS), Antwerp, Belgium; 6 Centre for Health Economics Research and Modelling Infectious Diseases (CHERMID), Vaccine and Infectious Disease Institute, University of Antwerp, Antwerp, Belgium; 7 Immunology Unit, Institut Pasteur du Cambodge, Pasteur Network, Phnom Penh, Cambodia; 8 Epidemiology and Public Health Unit, Institut Pasteur du Cambodge, Phnom Penh, Cambodia; 9 Virology Unit, Institut Pasteur du Cambodge, Pasteur Network, Phnom Penh, Cambodia; 10 Department of Paediatrics, Antwerp University Hospital, Antwerp, Belgium; 11 Unit of Ecology and Emergence of Arthropod-Borne Pathogens, Institut Pasteur, Paris, France; 12 Department of Biomedical Sciences,University of Antwerp, Antwerp, Belgium

**Keywords:** CCD, CD4+ T cells, chikungunya virus, CHIKV, chronic chikungunya disease, TCR, TCR repertoire, arthritogenic alphavirus

## Abstract

**Introduction:**

Chronic chikungunya disease (CCD) is characterized by persistent inflammatory joint pains following acute chikungunya virus (CHIKV) infection in about half of the patients . CD4^+^ T cells have been implicated in CCD pathogenesis, yet disease-associated T cell receptor (TCR) signatures remain undefined.

**Methods:**

Peripheral blood CD4^+^ T cells were collected from 65 Cambodian participants six months after RTqPCR–confirmed CHIKV infection during the 2020 outbreak, including chronic (n=16), non-chronic (n=16), and control (n=33) individuals. TCR α, β, γ, and δ CDR3 regions were sequenced and clustered using ClusTCR. Differential enrichment was assessed by Fisher’s exact test. L1-regularized logistic regression incorporating age, gender, and TCR clone counts was used to identify non-redundant TCR signatures.

**Results:**

Eight TCR clusters were differentially enriched between chronic and non-chronic patients. Chronic disease was associated with increased TRAV9-2 and TRAV41.2 and decreased TRAV41.3 and TRBV18 clone counts. Female controls exhibited higher baseline TRAV9-2 frequencies, suggesting a pre-existing, female-biased immune background associated with CCD susceptibility.

**Discussion:**

A distinct CD4^+^ TCR signature detectable six months post-infection characterizes patients who develop CCD. The association of TRAV9-2 with chronic disease and its enrichment in females suggests an underlying immune predisposition rather than persistent virus-driven expansion. These findings support a role for CD4^+^ T cells in CCD pathophysiology and identify candidate TCR-based biomarkers for disease risk stratification.

## Introduction

Chikungunya virus (CHIKV) is a mosquito-transmitted arthritogenic alphavirus. Related to the more endemic Ross River virus (Australia) and O’nyong nyong virus (sub-saharan Africa), CHIKV, originally described in East Africa, has expanded its geographic range over the past two decades to include Asia, Indian and Pacific Ocean islands, and the Americas and southern Europe (1–3).

Acute CHIKV infection typically presents with high viremia, fever, headache and rash. Most patients experience severe musculoskeletal and arthritic pain in large and small joints which usually resolves in 7–10 days following the rapid clearance of the virus by the immune system (4, 5). However, about 45% of patients progress to a chronic chikungunya disease (CCD) characterized by sustained joint pains that can last for months or even years (5, 6). This chronic post-viral arthritis, which clinically resembles rheumatoid arthritis (RA), represents a significant cause of disability and decreased quality-of-life in endemic regions and epidemic outbreaks in immune-naïve populations come with a considerable economic burden due to a crippling of the work force, accumulation of medical costs and the straining of healthcare systems (7, 8).

The cause and disease mechanisms of the sustained joint inflammation in CCD remain incompletely understood (9). Several explanations for the tissue inflammation, and the possible antigenic driver(s), during CCD have been put forward. These include the *de novo* presentation and/or increased accessibility of auto-antigens following joint tissue destruction and synovial membrane disruption and molecular mimicry, where exposure to viral antigens during acute infection can cause cross-reactivity to related auto-antigens (10, 11). Persistence of viral antigens in the joint tissue after peripheral clearance could also serve as a driver of prolonged inflammation (12). However, detection of residual RNA and viral protein in joint tissue and fluid in CCD patients has been highly inconsistent (13, 14). Like other chronic inflammatory (auto)immune diseases, CCD responds well to immunosuppressive therapies and similarly affects women decidedly more than men (15–19).

In humans there are marked parallels between CCD and RA patients in the associated cellular profiles in the peripheral blood (20, 21). And while in CCD the dynamics of immune cells in the affected joints has been studied mainly in mice, also here distinct similarities with RA pathogenesis have been described that include an influx of activated CD4+T, Th17, CD8+T and IFNγ producing NKT cells (22–25). Despite increased presence and activation of CD4+ T and CD8+ T cells in mouse models of CHIKV infection, neither cell type was found to be involved in the clearance of the virus (26, 27). In contrast, CD4+ T cells have emerged as important mediators of the sustained inflammation following CHIKV infection. In mouse models, CD4+ T cells are required for the development of arthritis-like symptoms, and depletion of these cells ameliorated joint inflammation (27). Similarly, human studies have found expansion of activated memory CD4+ T cells and a decrease in CD4+ regulatory T cells in CCD patients (28, 29).

T cell receptor (TCR) reactivity to (auto)-antigens drives the expansion of specific TCR signatures in activated-, effector memory- and or regulatory T cell compartments in infectious- or auto-immune diseases (30). Identification of TCR repertoires associated with a specific disease phenotype can shed light on the etiology of the disease (30, 31), supports identification of the antigenic driver (31, 32) and is and is increasingly used to derive biomarkers for diagnosis, prognosis, and monitoring of infection or vaccine responses (33).

To gain further insight into the physiopathology of CCD and to further the understanding of CD4+ T cell involvement we aimed to examine association of a specific TCR repertoire with CCD. To this end, we determined the TCR repertoire in bulk purified CD4+ T cells derived from peripheral blood from participants that did, or did not, progress to CCD, 6 months after confirmed infection during the 2020 CHIKV outbreak in Cambodia (34).

TCR clustering and regression analyses allowed identification of CD4+ TCR signatures specifically associated with non-chronic or chronic patients. A noted preferential presence of the TCR signature associated with chronic disease in female participants further suggests a gender-biased immune profile conducive to progression to CCD. Together these findings further support a role for CD4+ T cells in the etiology and pathophysiology of CCD and suggest candidate biomarkers that may predict susceptibility for developing chronic symptoms following CHIKV infection.

## Results

### Study participants

**Figure 1:**
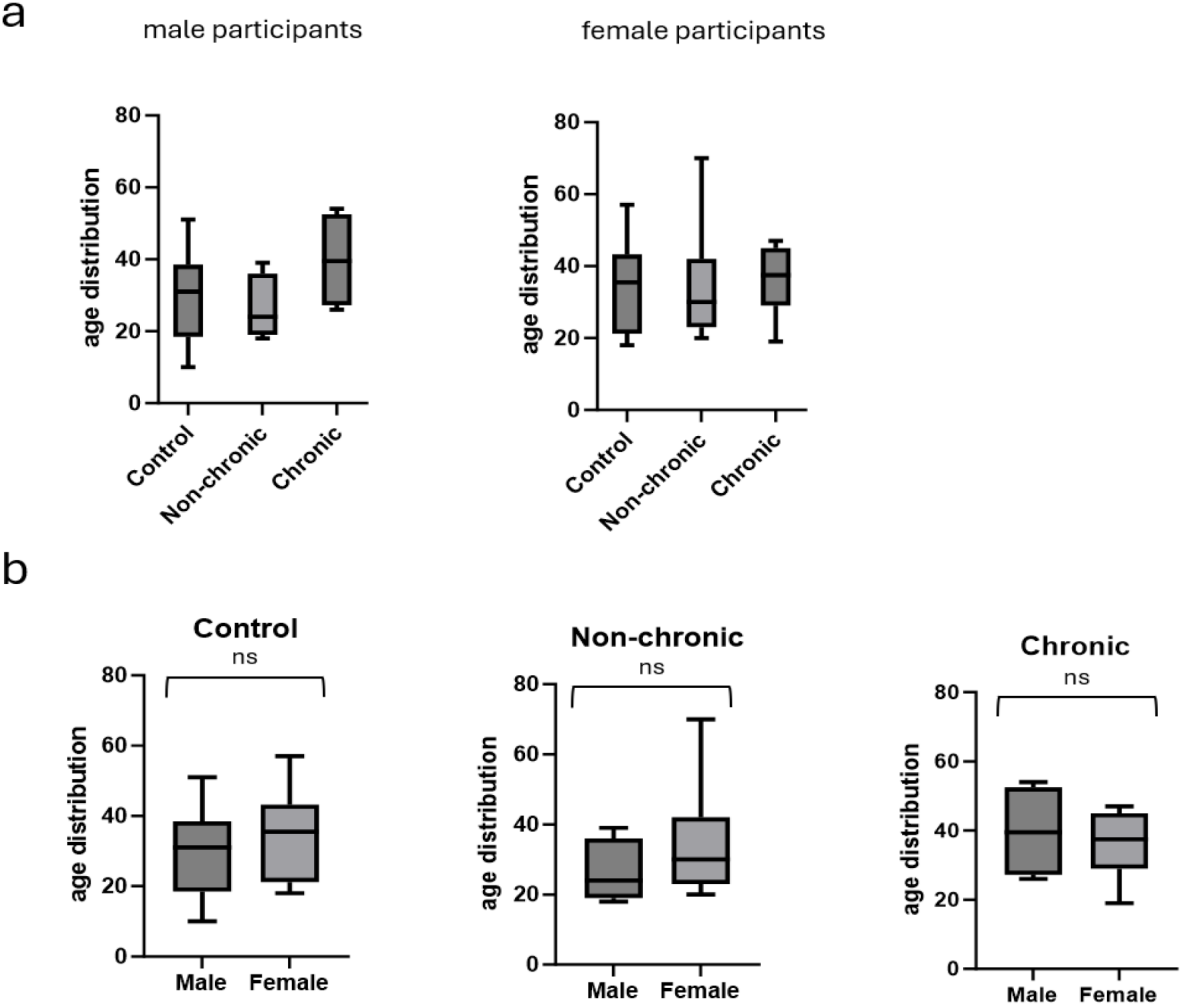
Number and age distribution of the participants across the groups in total and by gender. Box plots are median age and whiskers are min/max, **(a)** is ns Kruskall-Wallis non-para ANOVA (male p = 0.36; female p = 0.86) **(b)** is non-para T-test Mann-Whitney (control p = 0.268; non-chronic p = 0.31; chronic p = 0.58).

Following a nationwide chikungunya virus outbreak in Cambodia in 2020. peripheral blood CD4+ T cells were obtained from patients 6 months after a verified CHIKV infection. At the 6-month timepoint participants were clinically examined by the study nurse for presence or absence of persistent arthralgic symptoms (joint swelling, pain or redness). Non-infected, participants that did not present with arthralgia or reported previous arbovirus infections were included as controls. The participants were accordingly assigned to control, non-chronic and chronic groups. A total of 65 samples were obtained. **Table 1** summarizes the number and age distribution of the participants across the groups in total and by gender. No significant differences in age distribution were found between control, non-chronic and chronic groups within either gender (**Figures 1a, b**). Despite the small sample size in the male and chronic (n=4) group, this gender specific age distribution seems to reflect larger population data that establishes female gender as a risk factor for developing CCD at a younger average age than men (15–19, 35). Comparing average age between male and female participants in each group, there was no statistical difference in the control, non-chronic groups or chronic groups (**Figure 1b**).

**Table 1:** Summary of participants.

Category	Control	Non-chronic	Chronic
Number of Total Participants	33	16	16
Median Age (min/max)	32.0 (10-57)	29.0 (18-70)	37.5 (19-54)
Number of Male Participants (#)	13	5	4
Median Age (min/max) (Male)	31.0 (10-51)	24.0 (18-39)	39.5 (26-54)
Number of Female Participants (#)	20	11	12
Median Age (min/max) (Female)	35.5 (18-57)	30.0 (20-70)	37.5 (19-47)

### TCR cluster enrichment in non-chronic vs chronic patients

**Figure 2:**
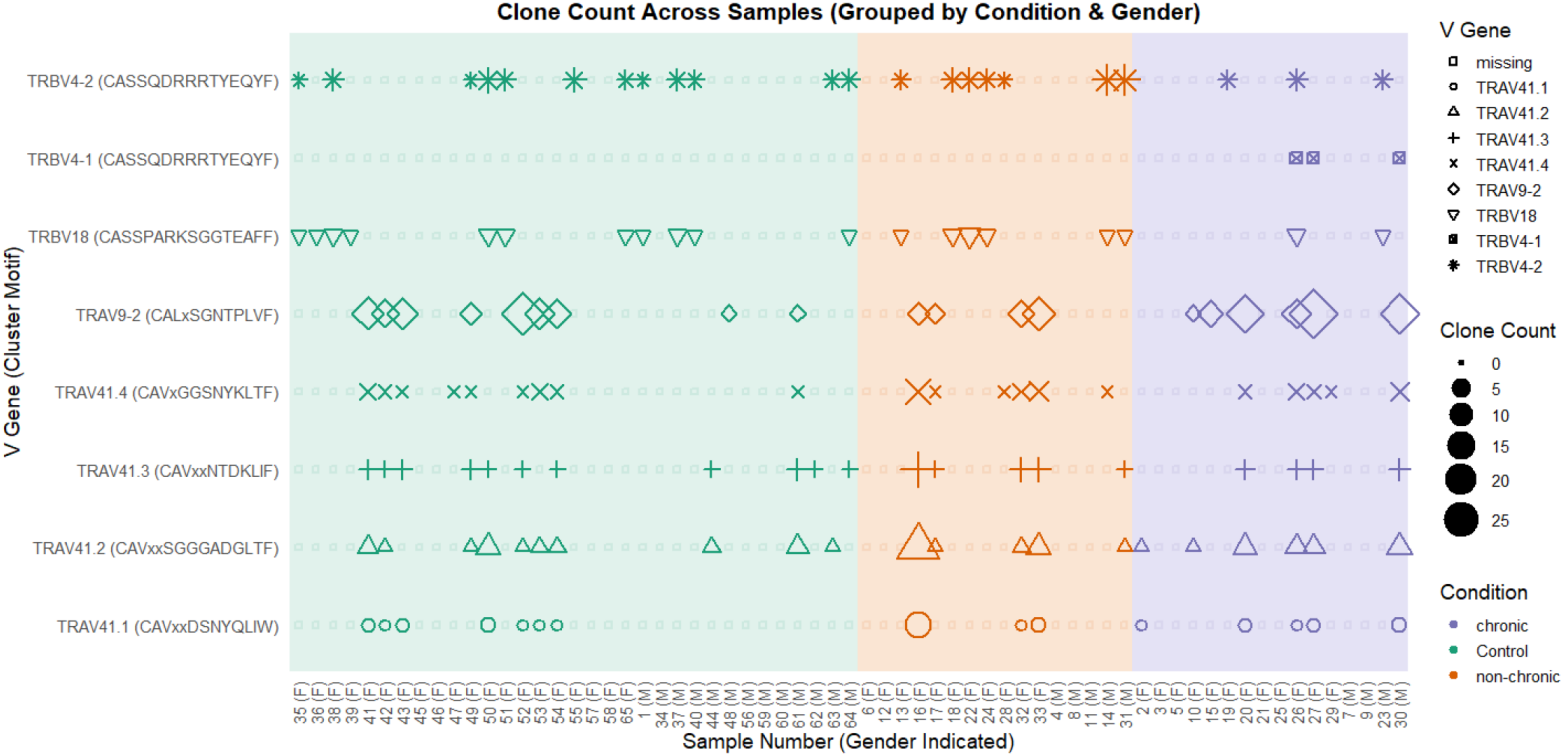
CD4+ T cell TCR clusters enriched in chronic or non-chronic patients. TCR clusters were determined in peripheral blood CD4+ T cells of patients that did (chronic) or did not (non-chronic) suffer from arthritic joint pains 6 months after verified CHIKV infection and in controls. CD4+ T cell clusters that were enriched in either chronic or non-chronic conditions are shown. Their presence in chronic, non-chronic and control patients are indicated. Clusters are indicated on the left with their consensus CDR3 motif. For each participant, gender is indicated at the bottom. Shape sizes indicate respective TCR clone counts. Participants are grouped by condition (green=control, orange=non-chronic, purple=chronic) and by gender.

**Figure 3:**
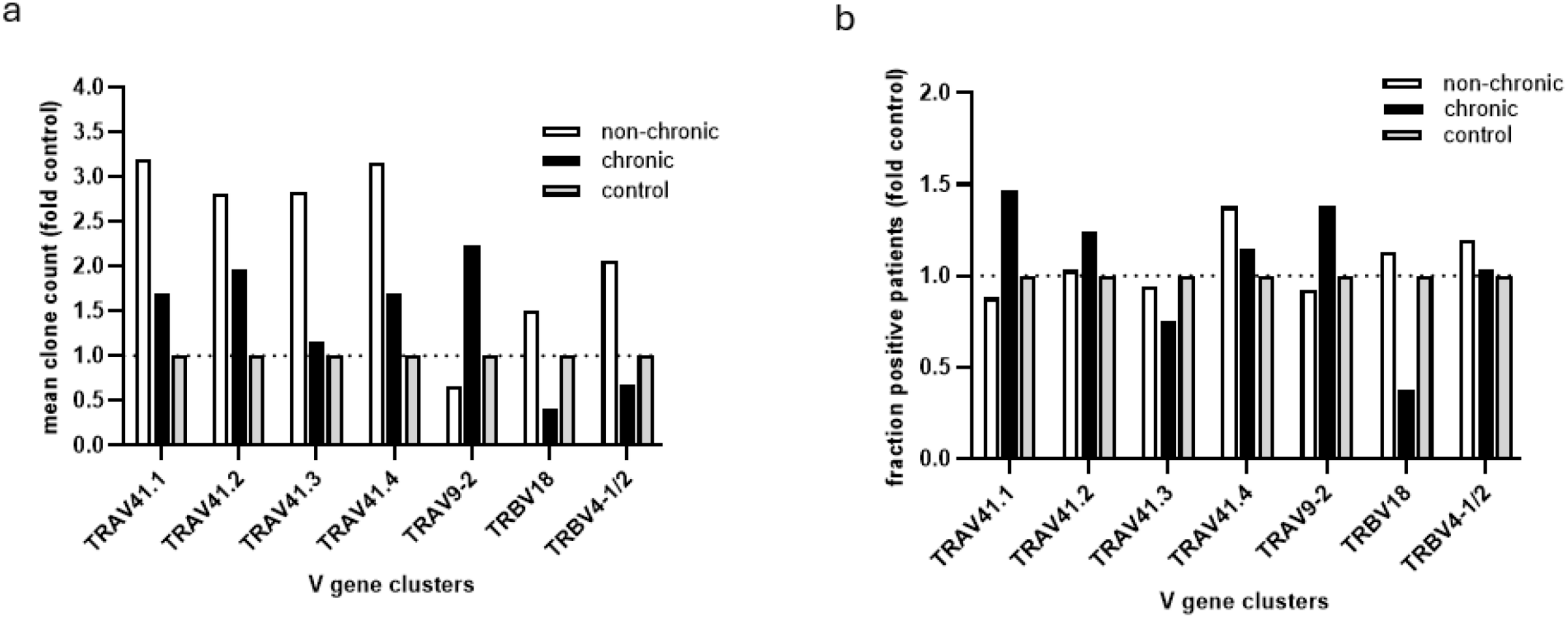
Comparison of prevalence and mean clone count of enriched TCR clusters in non-chronic and chronic condition to the control condition. **(a)** the fraction of participants in each condition in which the chronic or non-chronic associated TCR V-gene clusters could be detected (clone count > 0). Values were normalized for the calculated fraction in the control participants, which was set to 1. **(b)** mean clone count for each identified TCR V-gene cluster in each condition. Values were normalized for the mean clone count in the control participants, which was set to 1.

To assess whether the expansion of specific CD4+ TCRs is linked to the presence or absence of chronic arthritic symptoms following CHIKV infection, we analyzed the CDR3 regions of TCR α, β, γ, and δ chains in bulk-purified peripheral blood CD4+ T cells from control, non-chronic, and chronic patients. CDR3 repertoire clustering was performed using ClusTCR (36) and two-tailed Fisher’s exact test was applied to identify differentially modified TCR clusters in the chronic versus non-chronic condition (**Supplementary Figure 1**). At this stage, we purposely refrained from filtering out TCR clusters associated with other autoimmune- or infectious diseases, or those present in control patients, as this would preclude the identification of TCR sequences shared with diseases or previously present and potentially contributing to progression toward chronic disease. Eight TCR clusters were identified that were differentially present in the non-chronic or chronic condition. Seven TCR clusters associated with the non-chronic condition (four TRAV41 α-chains presenting four separate CDR3 motifs, a TRBV4–1 and TRBV4-2 β-chain containing an identical CDR3 motif and a TRBV18 β-chain), while one TCR cluster was more prevalent in patients with chronic symptoms (a TRAV9-2 α-chain) (**Figures 2**, **3**).

Comparison of average TCR cluster clone counts in the CHIKV infected conditions with those in control participants suggested that the TRAV41.1–4 TCR clusters had expanded in non-chronic participants, while less so in the chronic participants (**Figure 3a**). This while the TRBV18 and TRBV4-1/2 TCR clusters seemed to have retracted in the chronic participants while slightly expanded in the non-chronic condition (**Figure 3a**). In contrast, the average clone count in the TRAV9–2 TCR cluster increased in the chronic condition while remaining relatively unchanged in non-chronic participants compared to controls (**Figure 3a**). However, none of the modified TCR cluster motifs, identified by comparing non-chronic and chronic patients, were exclusive to either condition and representative clones were also readily detected in multiple control participants (**Figure 2**). Furthermore, for most of the TCR clusters, the portion of participants in which they were present did not substantially differ between the non-chronic, chronic and control groups (**Figure 3b**). Exceptions were an increase in prevalence of TRAV9–2 and TRAV41.1 and a decrease in TRBV18 in chronic patients.

The expansion and/or retraction of specific TCR clusters in non-chronic or chronic conditions compared to each other and control participants suggests that a differential CD4+ T cell reactivity is associated with chronic or non-chronic resolution of the CHIKV infection. However, while previous CHIKV infection in control participants was not ruled out unequivocally, together with the ready detection of the TCR clusters in the control participants, these observations suggest that CD4+ T cell stimulation by virus-derived-antigen-, or by *de novo* auto-antigen following CHIKV infection, and tissue damage, either do not underlie progression to chronic symptoms or simply wasn’t detected in peripheral blood derived CD4+ T cells in this study.

Consensus CDR3 amino acid motifs were determined for the enriched V-gene clusters (**Supplementary Figure 3**, and indicated in **Figure 2**) and used to perform homology- and antigen binding specificity searches with the TCRex tool (model version 2023-06-26 (37)) or exact matching against the VDJdb database (https://vdjdb.cdr3.net/credits). No high confidence homologies to previously reported CDR3 TCR chains or epitope binding prediction were found. Given the scope of these databases this finding shows that no known pathogen-induced (ie. CMV, TB, influenza, SARS-CoV-2, HIV-1) or self-antigen-induced TCRs were found associated with progression to- or protection from CCD. However, further manual literature curation and BLAST homology searches did however suggest involvement of certain TCR V-gene CDR3s identified here in related immunopathologies and are described in the discussion section.

### A CD4+ T cell TCR signature within patients that develop chronic symptoms after CHIKV infection

To determine which specific CD4+ TCR clusters and patient demographics associate most with persons that develop chronic symptoms, as a tool for selection of features for further analyses and as a redundancy detection tool, we performed logistic regression analysis comparing non-chronic and chronic patients. Age, gender and clone counts of the different TCR V-genes were considered as predictive features. L1 regularized logistic regression was chosen to prevent feature inflation by discounting redundant predictors and overfitting of the model due to the relatively small sample size. Despite the exclusive association of TRBV4–1 with the chronic condition in **Figure 2** we chose to combine TRBV4–1 and TRBV4–2 clusters in subsequent analyses given their identical CDR3 motif and thus presumed shared antigen binding specificity and stimulation.

**Figure 4:**
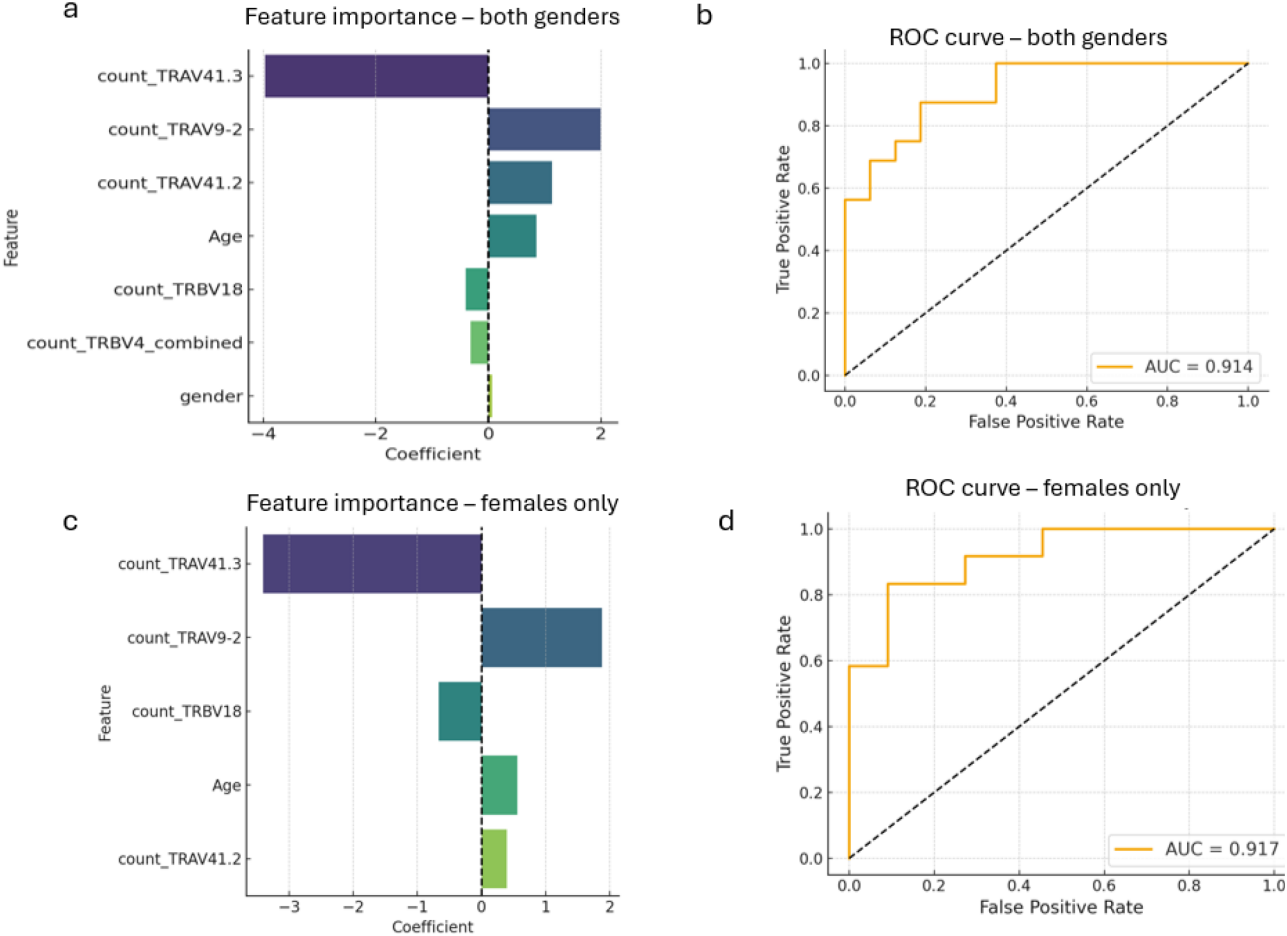
Regression analysis allows definition of CD4+ TCR signature associated with chronic CHIKV disease (CCD). L1 regression analysis was performed taking into account age, gender and clone counts in the TCR clusters that were found enriched or depleted in chronic versus non-chronic patients. Bidirectional histograms indicating determining features and regression coefficients for the **(a)** all-participant and **(c)** female-only regression models are shown. Corresponding predictive performances of the regression models are shown in ROC curves in **(b)** and **(d)** and AUCs indicated.

Including both male and female participants, a regression model (ROC curve **Figure 4b**, AUC = 0.91) suggested that, in line with previous studies, increased age (coeff +0.857) was predictive of developing chronic symptoms (**Figure 4a**). Female gender, while associated with the chronic condition (coeff +0.064), was only a minor predictor (**Figure 4a**), possibly due to a limited number of male participants in the non-chronic and chronic conditions. Importantly, as the main predictive features, an increase in clone count of the TCR TRAV41.3 cluster (coeff -3.964), and to a lesser degree the TRBV4 (coeff -0.318), were associated with the non-chronic condition. While increased presence of TRAV9-2 (coeff +2.0) and TRAV41.2 (coeff +1.125) and a decrease in TRBV18 (coeff -0.399) clusters characterizes patients that develop chronic symptoms (**Figure 4a**).

Chronic inflammatory autoimmune diseases are significantly more frequent in women compared to men. To assess whether this is reflected in a gender-specific TCR signature associated with CCD we performed the same regression analysis for all genders separately. Unfortunately the limited number of male participants in the chronic (n=4) and non-chronic (n=5) condition were too few to allow confident regression modeling. However, a confident regression model could be built comparing females between the non-chronic (n=11) and chronic conditions (n=12) considering age and clone counts of the different TCR clusters as predictive features (ROC curve **Figure 4d**; AUC = 0.917). While the proportionate weights of some predictors changed slightly, the overall association of the different TCR V-genes remained the same with strongest association of TRAV41.3 with the non-chronic condition (coeff -3.4) and an increase of TRAV9–2 and a decrease of TRBV18 in women that develop chronic conditions (coeff +1.87) (**Figures 3**, **4c**).

These findings are noteworthy as we were able to identify a TCR signature in peripheral blood-derived CD4+ T cells, 6 months after an initiating CHIKV infection, that characterizes patients who did or did not develop a chronic inflammatory disease that is mainly localized to the joints. The finding is further supportive of an increasingly suggested role for CD4+ T cells in the ongoing inflammatory pathology of CCD in humans (38–40).

### Female-associated TCR signatures in controls suggests a gender-biased immune background associated with progression to CCD

**Figure 5:**
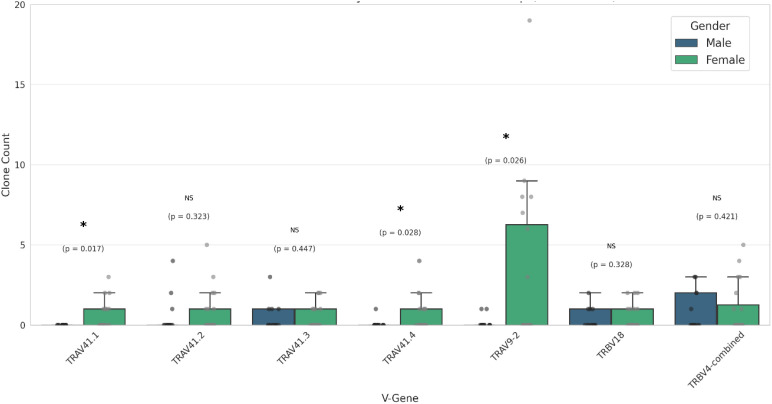
Clone count distribution of TCR clusters by gender in the control condition. Median clone counts of the different TCR clusters (denoted by their V-gene) are shown by gender for the control condition participants. Median clone counts are shown, error bars indicate 95%CI. P-values are indicated (* indicates P-value< 0.05; Welch’s T Test).

Given the increased risk of females to progress to CCD as compared to men, we investigated whether a gender-specific immune signature related to disease progression could be detected in the female control group participants. Indeed, comparing TCR cluster clone counts between male and female control participants we found a significantly higher presence of TRAV41.1, TRAV41.4 and TRAV9-2 (**Figure 5**) in female control participants, the latter of which was identified as a main predictor for chronic disease in both the female-only and all-participants regression models (**Figure 4**).

This finding suggests the existence of an immune background signature, characterized by an increased presence of specific TRAV9–2 TCR clones that is more prevalent in females and which might have predictive value for risk of developing CCD.

## Discussion

Following a self-limiting acute febrile phase, CHIKV infections lead to a debilitating arthralgia in major and minor articulations of the upper and lower limbs that can last 3 months up to several years in an estimated 40% of patients (5, 41). While the pathophysiology and clinical presentation of CCD can resemble those of rheumatoid arthritis, the etiology and antigenic driver(s) of the chronic inflammation of the joints remain to be determined (9, 20, 41). Earlier studies in murine- and non-human primate models identified CD4+ T cells as a driver and modulator of chronic CHIKV arthritis (27, 41) and an increasing body of human data corroborates their role in the disease process (38–40). Characterizing disease-associated T cell receptor (TCR) repertoires can clarify the involvement of T cells in immunopathologies, possibly allow identification of pathology-driving antigens and develop diagnostic or prognostic biomarkers.

In this study, we examined the T cell receptor (TCR) repertoire in peripheral blood-derived CD4+ T cells from patients with chronic joint pains six months post-CHIKV infection and from individuals who recovered without chronic symptoms. In addition a, regionally matched, control group was included who did not report joint pains nor previous arbovirus infections. CD4+ T cells were bulk purified from peripheral blood and TCR variable chain mRNA was sequenced. Variable CDR3-region amino acid sequences were used to perform TCR cluster analysis which assumes shared antigenic epitope recognition by related variable chain TCR sequences. Comparing overall presence and size of TCR clusters between chronic and non-chronic patients we identified several TCR alpha- and beta- chains who were differentially present in either condition and seemed to characterize patients that developed chronic joint pains following an initiating CHIKV infection.

As none of the TCR cluster V-genes were exclusively present in either condition, no single marker stood out on its own towards delineating a patient’s condition. However, considering combinations of the differentially present TCR clusters by regression modeling highlighted the more determinant TCR V-genes characterizing patients with chronic symptoms. Independent of gender, a marked increase in TCRs carrying a TRAV9–2 alpha chain was found in chronic patients who are also characterized by a relatively low presence of TRAV41.3 alpha chain carrying CD4+ T cells.

The TCR sequencing procedure applied here was limited to the CDR3 region of TCR chains. While allowing bioinformatic TCR clustering analyses this approach does limit the potential for further in-depth molecular analysis of intact TCRs and their antigen binding specificity. Similarly, our bulk sequencing approach further precludes identification of coexpressed TCR chains. Given the involvement of both chains in antigen epitope binding this further limits the potential for identification of the specific antigens driving the differential presence of the TCRs in the different patient groups. Future identification of the specific TCR chain pairs associated with chronic disease through single-cell TCR sequencing should allow for more in-depth characterization of antigenic epitope reactivity of the identified TCR alpha chains.

As we also detected the chronic disease-associated TCR V-gene clusters at comparable levels in a good number of presumably uninfected controls, we consider that these TCRs do not derive from uniquely CHIKV reactive CD4+ T cells, nor from CD4+ T cells actively reacting to CCD-associated auto-antigens that are newly presented following coincident joint tissue damage. However, the differential increase in TRAV9–2 in patients with a chronic inflammatory disease does suggest a reactive activation of these CD4+ T cells following CHIKV infection and a possibly a role in the chronic inflammatory pathology, while the decrease of TRAV41.3 in chronic patients suggests a decreased activation in patients that develop chronic symptoms, possibly suggesting that proper stimulation of TRAV41.3 could represent a protective immune signature.

The absence of CHIKV-specific CD4+ T cell TCRs is seemingly at odds with a recent finding of increased numbers of CHIKV-specific CD4+ T cells in the peripheral blood of CCD patients (38, 39) as determined by phenotyping of activated cells following exposure to CHIKV-derived epitopes. We therefore have to consider that our CD4+ T cell purification- and sequencing approach was not sufficiently sensitive to detect sparse CHIKV-specific TCRs, whose clonal expansion during acute infection would have retracted into the memory T cell compartment over the 6-month period between infection and clinical evaluation of chronic disease. As CHIKV-specific CD4+ T cells were found only in CD4+ T memory subsets, that there is no indication of sustained viral replication driving the chronic joint inflammation (38) would however be supported by the absent detection of CHIKV-specific TCRs in this study, as this suggests an absence of ongoing, reactive, CD4+ T cell expansion against CHIKV-derived epitopes.

L1 linear logistic regression analysis minimizes feature inflation by discounting redundant predictors and overfitting of models for relatively smaller sample sizes. Here it was performed to identify the most discerning V-gene clusters and demographic characteristics for further analysis by comparing between chronic and non-chronic patients, taking into account clone counts of the identified TCR V-genes as well as age and gender. As bootstrap resampling indicated model stability (**Supplementary Figure 4**), label permutation tests showed little collapse to an AUC of 0.5 (**Supplementary Figure 4**) as would be expected. As various reasons for this could exist, the relatively small sample size of this study is expected to be a contributor and future studies should aim to include more participants. Nevertheless, the determined AUC for the models did approach, and reach, statistical significant separation from the null AUC for the all-participant model (p=0.058) and female-only model (p=0.043), respectively. As indeed confidence in relative feature importance in discriminating chronic and non-chronic patients through these models is thus limited, they do not allow confident prediction of chronic disease or be useful for clinical decision making for example. However, as enrichment analyses in the initial comparison between chronic and non-chronic patients identified statistically significant associations of separate TCR clusters with CCD, the use of the regression models as a feature-selection-, and association- and redundancy detection tool, for subsequent focus on specific TCR clusters for further analysis, is justified.

In line with many previous observations, increased age associated with chronic disease status (35). Surprisingly, in the mixed-gender regression model, female gender was not a determinant factor for chronic disease as consistently observed in CCD studies. This incoherence was however subsequently explained by the finding that the most correlated factor with chronic disease, TRAV9–2 V-gene count, strongly associates with female gender in this cohort, rendering female gender a largely redundant feature. Identification of a discerning TCR V-gene signature with a limited number of TCR V-genes (relatively low TRAV41.3 and TRBV18 and relatively high TRAV9–2 and TRAV41.2 V-gene count) for CCD patients further suggests the presence of disease-antigen specific TCRs that can support development of chronic inflammatory disease after CHIKV infection.

While possible, the absence of clear TCR clonal expansion after CHIKV infection, compared to non-infected controls, could be equally suggestive of having detected representative TCRs of a background immune state that supports or protects against chronic progression. In this light a genetic determining factor such as HLA-biased VDJ selection, other immune functionalities or TCR V-gene selection determining factors such as different external exposures to antigen or allergens or innate immune responses could be explanatory for the association of certain TCR clusters with progression to CCD. This seems supported by the finding here that the chronic disease-associated TCR V-genes were preferentially detected in females. Indeed resembling the common predisposition of women for developing chronic inflammatory (auto) immune pathologies for which the contributions of various such factors are similarly still debated (42).

Interestingly, despite too few male participants having been included in the non-chronic and chronic conditions to allow regression modeling, an increased TRAV9–2 cluster also seemed to associate with chronic patients from the male gender. None of the male participants (0 of 5) in the non-chronic condition had detectable TRAV9–2 while 1 in 4 male patients with chronic symptoms had remarkably higher levels of TRAV9-2 (clone count = 15) than those (2 of 13) in the control group (clone count = 1).

Apart from highlighting non-redundant associations of TCR clusters prevalence with chronic disease, the regression models were suggestive of disease status based on this limited set of predictive features (**Supplementary Figure 4**: ROC curves for both models) in this cohort. Given the non-prospective nature of the study, which precludes establishing a causal relation between preexisting immune status, as characterized by specific TCR signatures and progression to CCD, this finding in itself holds little diagnostic or prognostic value. However, comparison with the non-infected controls allows at least some temporal inference of disease progression and immune profile and suggests that the chronic disease associated TCR signature is already present in a subset of patients before the initial infection and could possibly predict stratification into patients that do or do not develop chronic symptoms after infection. Future prospective studies could verify and validate this TCR V-gene signature as a potential biomarker that could have prognostic value for progression to chronic disease and support decisions in medical care for patients at risk.

TCR CDR3 searches using the TCRex tool or matching against the VDJdb database did not return any suggestions of epitope binding or involvement in other pathologies for the TCR chains identified here. This finding is strongly suggestive of the identified TCRs not having been expanded or modulated by exposure to common infectious disease such as flu, cytomegalovirus, tuberculosis and SARS-CoV-2. More basic BLAST homology- and manual literature searches did however turn up involvement of TRAV9–2 clonotypes, and their CDR3 motifs as identified here in CCD (CALxSGNTPLVF), in other (auto)immunopathologies. Increased TRAV9–2 clonotype TCRs are for example found in celiac disease, a chronic inflammatory disease mediated by T cells (43) or in the recognition of self-phospholipids presented by CD1b (44). In active and disseminated tuberculosis, glycolipid-specific T cells increasingly express TRAV9–2 among other TCRs (45).

TRAV9–2 presenting CD4+ T cells seem to be an integral part of the immunopathology of Ni2+ allergy (46–48) as TRAV9–2 clonotype TCRs are strongly, and specifically, induced by nickel treatment (48). Given the positive correlation between nickel exposure and the development of arthritis (49), and the comparable female gender and age correlatives with nickel-allergies, rheumatoid arthritis and CCD, we wondered whether any of the specific TRAV9–2 CDR3s we found associated with CCD were also identified in nickel responsive CD4+ T cell TRAV9–2 clonotypes. Interestingly, we found three TRAV9–2 cluster member CDR3s associated with CCD (CALNSGNTPLVF, CALRSGNTPLVF and CALTSGNTPLVF) in nickel-stimulated, but not unstimulated cells (MiXCR TCR analysis (50) of raw RNASeq data available from Siewert lab publication (48) and identity search against our TRAV9–2 CDR3s was performed). This suggest the possibility of an overlap of immune states that are conducive to developing nickel allergies, rheumatoid arthritis and arthritic disease in CCD. This would be in line with the fact that females are indeed more prone to developing nickel allergies (46), either or not due to increased jewelry use, as they are to developing rheumatoid arthritis as well as CCD. It would be of interest in future studies to fully assess allergies and/or allergic sensitivities and exposures to metals and other compounds for their correlation with development of CCD.

A more direct link between TRAV9–2 and other joint related immunopathologies was also suggested by the finding of overrepresented TRAV9–2 clonotype carrying CD4+ T cells in synovial tissue biopsies from rheumatoid arthritis patients (51). Furthermore, while not identical, related CDR3 regions compared to the ones identified here as associated with CCD, were similarly found in synovial tissue derived T cells from rheumatoid arthritis patients, while absent from control patients in this study (52) (comparing CALxSGNTPLVF from this study to CAVRDLSGNTPLVF and CAVHSGNTPLVF from (52)).

## Materials and methods

### Patient samples

Heparinized blood samples were obtained from 65 volunteers enrolled in the CHRONICHIK study 6 months after a RT-qPCR confirmed CHIKV infection during the 2020 outbreak in Cambodia (34). Regional CHIKV negative volunteers were included as controls. Between the initial diagnosis and sampling at 6 months, the volunteers did not report to be diagnosed with DENV, ZIKV or other febrile or arthritogenic infections. At the 6 month timepoint the volunteers were examined for presence or absence of clinical signs and symptoms of chronic arthritis by the study nurse. All joints were evaluated for redness, swelling and pain. In case at least 1 join showed persistent symptoms, the individual was classified as a CCD case, whereas patients with absence of joint symptoms were classified as non-chronic. Peripheral blood mononuclear cells (PBMC) were isolated using density gradient centrifugation, cell viability was assessed by trypan blue exclusion, cells counted, resuspended in 10% of dimethyl sulfoxide (Sigma-Aldrich) and 90% of fetal bovine serum (FBS) (Thermo Fisher Scientific) and stored in liquid nitrogen until analysis. Ethical approval for the study was granted by the National Ethics Committee for Health Research Cambodia (NECHR #2020-313) and the Institutional Review Board of the Institute of Tropical Medicine, Antwerp, Belgium (IRB/AB/AC/228_1453/20). Total and gender- and age- categorized participant numbers are summarized in **Table 1**.

### CD4+ T cell purification and mRNA isolation

Cryopreserved PBMCs were thawed at 37 °C and dropwise resuspended in 1ml, 37 °C, thawing medium [RPMI 1640 (Biowest), 20% FCS (Gibco), 3U/ml rec. RNAse-free DNAse I (ThermoFisher)]. Cells were rinsed twice by centrifugation at 400 g for 10 min and resuspension in 5 ml thawing medium. Viability was assessed by trypan blue exclusion and 1.10^6^ viable cells/ml left to rest overnight in culture medium [RPMI, 10% FCS]. The next day viable cells were recovered using the dead cell removal kit (Miltenyi Biotec, 130-090-101) according to the manufacturer’s specifications and subsequently CD4+ T cells were positively selected using CD4 MicroBeads (Miltenyi Biotec, 130-045-101) according to the manufacturer’s specifications and counted and viability determined by trypan blue exclusion. Purity of the CD4+ T cell population and efficient removal of CD8 T cells was verified by fluorescence cell counting (FACSVerse, BD Biosciences) on 10.000 cells of select samples after paraformaldehyde fixation (1% PF, PBS, 10min, RT), washing and staining with anti-CD8-APC_eFLuor780 (Invitrogen, 47-0087-42), anti-CD4-BV421 (BD Horizon, AB_11154417) and anti-CD3-FITC (eBioscience, 11-0037-42). CD4+ T cells were washed and 20.000-150.000 cells frozen at -80 °C until mRNA isolation. Total RNA was purified using Quick-RNA MicroPrep kit (Zymo, R1050). Integrity of extracted RNA was verified using Tapestation RNA ScreenTape (Agilent).

### T cell receptor library preparation and sequencing

CDR3 regions of TCRα, -β, -γ, and -δ chains were identified using the QIAseq Immune Repertoire RNA Library kit (Qiagen, Venlo, The Netherlands) according to manufacturer’s instructions. Briefly, RNA of each bulk CD4+ T cell sample was divided into three technical repeats for the PCR based library prep. After quality control using TapeStation (Agilent, Santa Clara, CA), concentration was measured with the Qubit dsDNA HS Assay kit (Thermo Fisher Scientific, Waltham, MA) and pools were equimolarly pooled and prepared for sequencing on the NextSeq platform (Illumina, San Diego, CA) using the Nextera XT assay and v2 Index (Illumina, San Diego, CA).

### TCR repertoire clonal analysis

#### Preprocessing

raw sequencing data were preprocessed using MiXCR (v. 3.0.7 (50)). Further preprocessing and analyses were carried out under Python version 3.11.9. To ensure biologically plausible TCR rearrangements, CDR3 sequences generated by non-functional V and J gene were discarded based on the ImMunoGeneTics information system database (IMGT (53)). Additionally, CDR3 sequences that did not exhibit the canonical structural constraints, specifically, those lacking an N-terminal cysteine (C) and a C-terminal phenylalanine (F), were excluded from the analysis. Unique clonotypes were defined by the combination of their V-gene and amino acid CDR3 sequence, resulting in a total of 407721 unique clonotypes across the cohort (**Supplementary Figure 1**).

We found that TCR-assigned read counts spanned around 102–105 per sample and we did not find significant differences between control, non-chronic, and chronic groups, indicating that differential sequencing depth did not bias the between-group comparisons (**Supplementary Figure 1a**). Across all samples, 407,721 unique clonotypes were identified after stringent quality filtering, and clonal size distributions spanned rare to hyperexpanded clones without systematic group-specific skewing (**Supplementary Figure 1b**), indicating preserved repertoire homeostasis.

#### Clustering and enrichment testing

CDR3 repertoire clustering was performed using ClusTCR (v. 1.0.2 (36)). Clusters containing sequences derived from only a single individual (private clusters) were excluded from further analysis. To identify clusters enriched in either the chronic or non-chronic conditions, a two-tailed Fisher’s exact test was applied. Clusters were considered significantly enriched if the Benjamini-Hochberg adjusted – log10 p-value was below the standard threshold of 0.05 (**Supplementary Figure 2**). The contingency table of the test was thus constructed:

**Table:** Contingency table Fisher's exact test.

X	n−X
N−x	M − N − n + x

Where: x equals to the number of positive sequences in the cluster, M is the number of unique sequences in the cohort, n is the overall number of positive sequences and N is the size of the cluster.

To reduce overcorrection due to the large number of clusters, only those with an absolute log2 fold change greater than 1 were considered. The log2 fold change was calculated as:


$$log2\;(\frac{\left(\frac{number\;of\;positive-class\;clones\;in\;the\;cluster+pseudocount}{\;sum\;of\;positive-class\;clones\;in\;the\;cohort}\right)}{\left(\frac{number\;of\;negative-class\;clones\;in\;the\;cluster+pseudocount}{sum\;of\;negative-class\;clones\;in\;the\;cohort}\right)})$$

A pseudo-count of 0.01 was added to both positive and negative counts to prevent division by zero.

#### Statistical analysis

Logistic regression analyses were performed using Python3 (54). Data preprocessing and manipulation were conducted with the pandas library (55). V-gene clone counts were aggregated by participant, and TRBV4–1 and TRBV4–2 were combined into a single predictor. Binary logistic regression was implemented using scikit-learn (56) with L1-regularized variants. Predictive performance was evaluated via ROC curves and AUC scores. Feature importance was assessed based on absolute model coefficients. Robustness of the produced models was subsequently verified by bootstrap resampling (n=10000; **Supplementary Figure 4**). Label permutation tests for the all-participant- and female-only regression models were performed (**Supplementary Figure 5**).

## Data Availability

The datasets generated for this study have been deposited in the NCBI Sequence Read Archive (SRA) under BioProject accession number PRJNA1429370. The data are publicly available at: https://www.ncbi.nlm.nih.gov/bioproject/PRJNA1429370/.

## Supplementary material

Supplementary Figure 1 Sequencing depth and clonal proportions across samples and conditions. **(a)** boxplots showing total repertoire sequencing reads for control, non-chronic, and chronic samples. Interquartile ranges are indicated with horizontal lines denoting the median. Whiskers extend to 1.5x interquartile range. Differences between groups were assessed using a Kruskal–Wallis test (p = 0.496). **(b)** Stacked bar plots indicate the proportional distribution of clonotypes across five clonality groups for each individual sample. Clonotypes were classified as rare (0< X ≤ 10^-5^), small (10^-5^< X ≤ 10^-4^), medium (10^-4^ X ≤ 0.001), large (0.001< X ≤ 0.01), or hyperexpanded (0.01< X ≤ 1), where *X* represents clonal frequency within the repertoire. Samples are grouped by condition (control, non-chronic, chronic).

Supplementary Figure 2 CDR3 repertoire clustering and enrichment analysis in non-chronic vs. chronic condition. TCR cluster analysis was performed using ClusTCR and two-tailed Fisher’s exact test was performed to identify enriched TCR clusters in the chronic versus non-chronic condition.

Supplementary Figure 3 Consensus CDR3 amino acid motifs of the enriched TCR V-gene clusters. **(a–g)** Consensus CDR3 amino acid motifs were determined using Seq2Logo 2.0 (57) with Shannon logo type and without weight on priors.

Supplementary Figure 4 Bootstrap validation for all-participants and female-only of L1 regression models. Replacement bootstrapping was performed for both regression models with n=10.000 resampling iterations. For the all-participants model a mean AUC of 0.9513 was calculated with a 95% confidence interval of 0.8097-1.00. For the female-only model a mean AUC of 0.9627 was calculated with a 95% confidence interval of 0.7917, 1.00.

Supplementary Figure 5 Label permutation testing of L1-regularized logistic regression models. Label permutation testing of L1-regularized logistic regression models was performed for the **(a)** both gender regression model and **(b)** the female-only regression model. Outcome labels were randomly permuted 1,000 times and model performance was evaluated. Histograms show the distribution of AUCs obtained under label permutation; the vertical line indicates the observed AUC. The mean and standard deviation of the permuted AUCs and the permutation p-value is indicated.
