# Supplementary material for: Identification of a TCR signature in peripheral blood derived CD4+ T cells, associated with chronic chikungunya disease, suggests a conducive, female-biased, background immune profile
Source: Front Immunol. 2026 Mar 11;17:1739100. doi: 10.3389/fimmu.2026.1739100 (PMC13013352; doi:10.3389/fimmu.2026.1739100)
Supplement: Supplementary Figure 1 — Sequencing depth and clonal proportions across samples and conditions. (a) boxplots showing total repertoire sequencing reads for control, non-chronic, and chronic samples. Interquartile ranges are indicated with horizontal lines denoting the median. Whiskers extend to 1.5x interquartile range. Differences between groups were assessed using a Kruskal–Wallis test (p = 0.496). (b) Stacked bar plots indicate the proportional distribution of clonotypes across five clonality groups for each individual sample. Clonotypes were classified as rare (0< X ≤ 10-5), small (10-5< X ≤ 10-4), medium (10-4 X ≤ 0.001), large (0.001< X ≤ 0.01), or hyperexpanded (0.01< X ≤ 1), where X represents clonal frequency within the repertoire. Samples are grouped by condition (control, non-chronic, chronic). [file Image1.pdf]

Suppl Fig.1

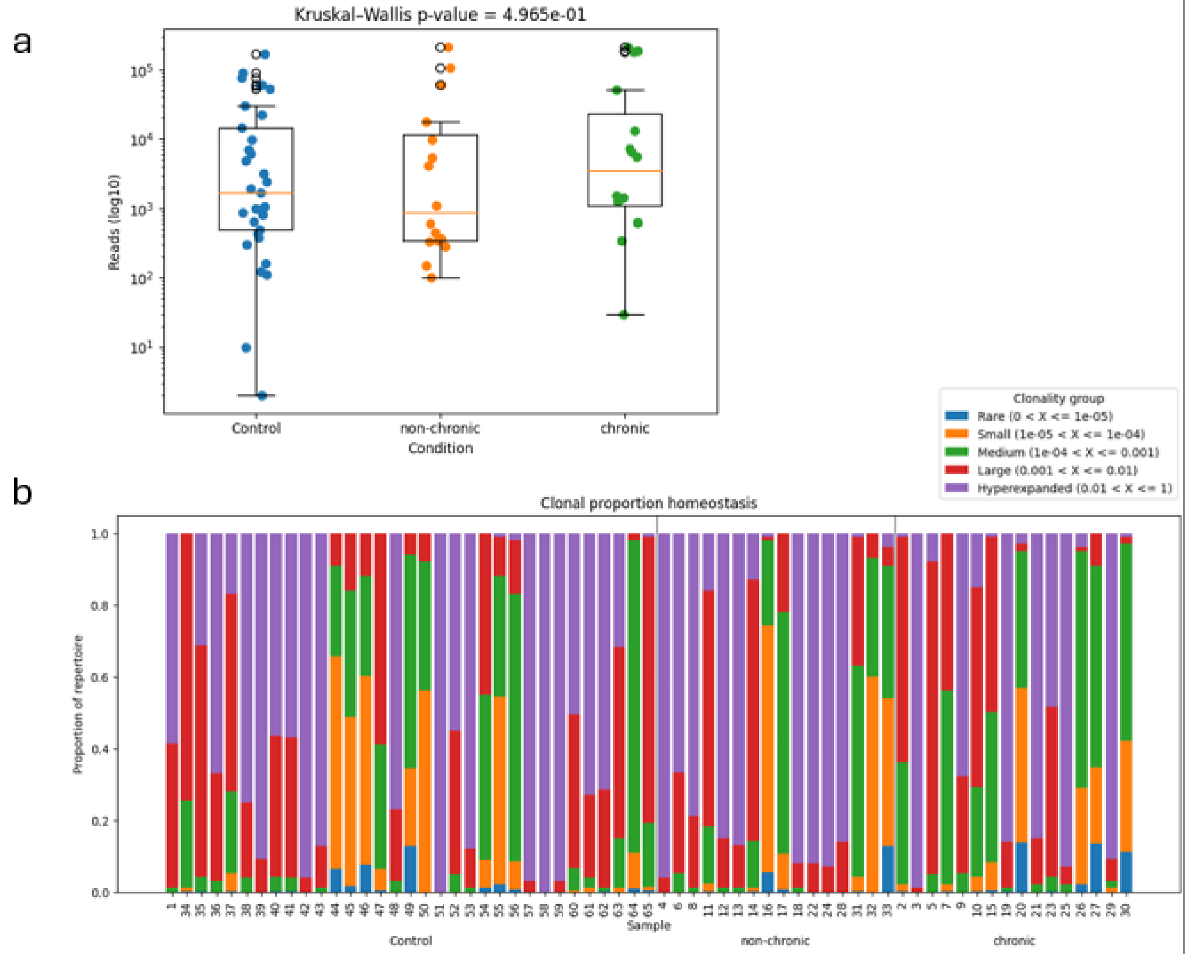

Suppl. Fig. 1 *Sequencing depth and clonal proportions across samples and conditions*

a) boxplots showing total repertoire sequencing reads for control, non-chronic, and chronic samples. Interquartile ranges are indicated with horizontal lines denoting the median. Whiskers extend to 1.5x interquartile range. Differences between groups were assessed using a Kruskal–Wallis test ($p = 0.496$). b) Stacked bar plots indicate the proportional distribution of clonotypes across five clonality groups for each individual sample. Clonotypes were classified as rare ($0 < X \leq 10^{-5}$), small ($10^{-5} < X \leq 10^{-4}$), medium ($10^{-4} < X \leq 0.001$), large ($0.001 < X \leq 0.01$), or hyperexpanded ($0.01 < X \leq 1$), where *X* represents clonal frequency within the repertoire. Samples are grouped by condition (control, non-chronic, chronic).

Suppl Fig.2

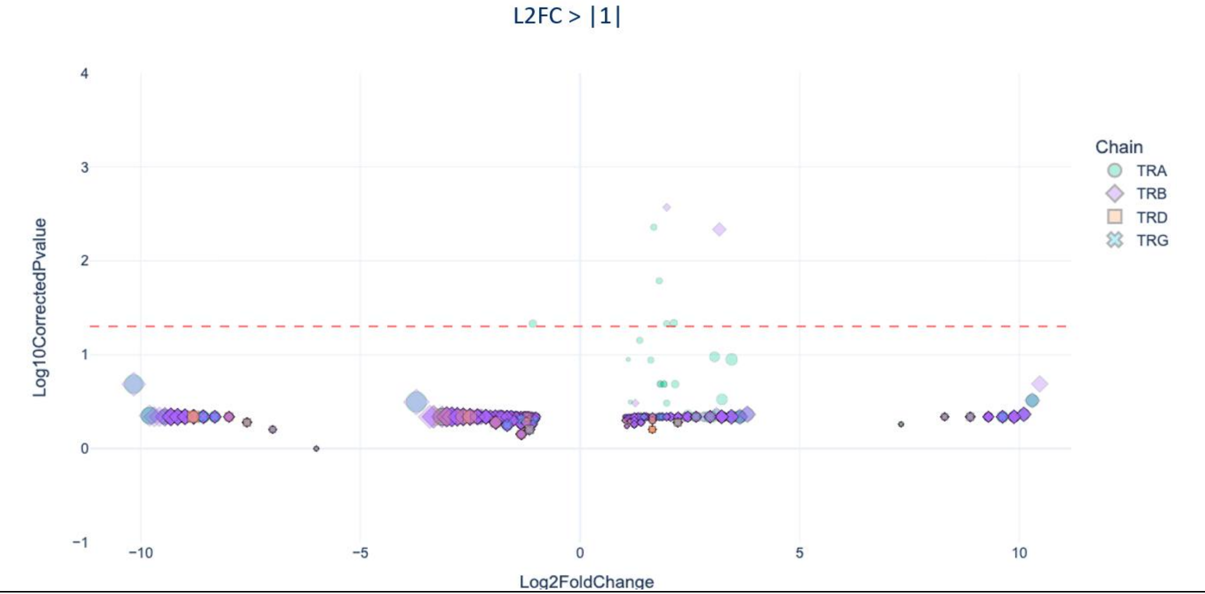

Suppl. Fig. 2 *CDR3 repertoire clustering and enrichment analysis in non-chronic vs. chronic condition*.

TCR cluster analysis was performed using ClusTCR and two-tailed Fisher's exact test was performed to identify enriched TCR clusters in the chronic versus non-chronic condition.

Suppl Fig.3

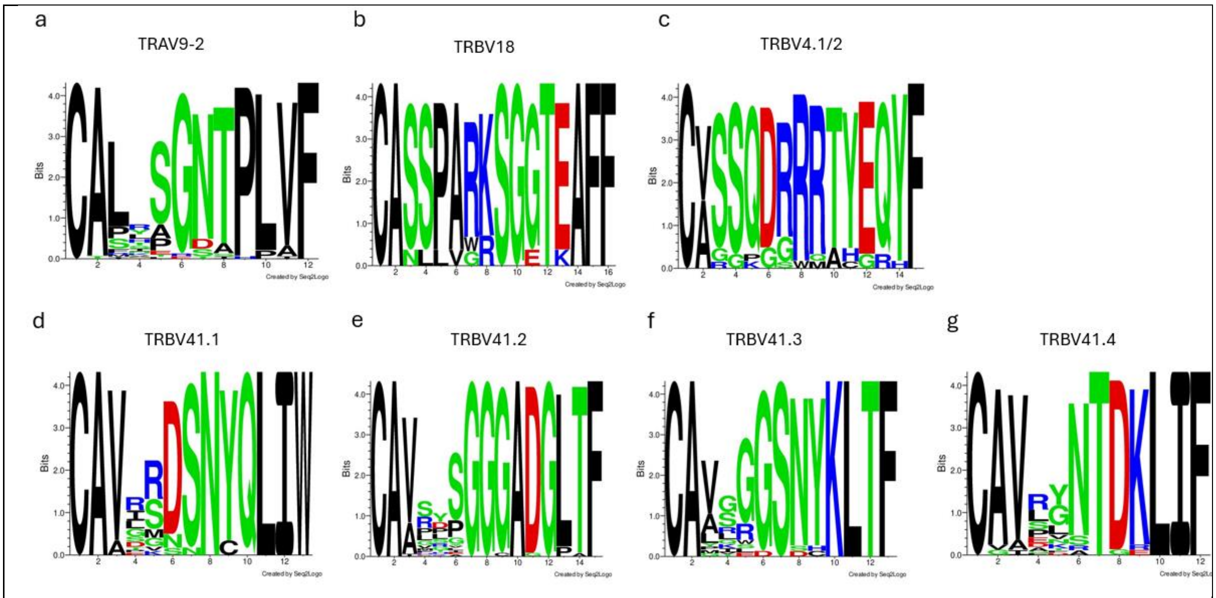

Suppl. Fig. 3 *Consensus CDR3 amino acid motifs of the enriched TCR V-gene clusters*

(a-g) Consensus CDR3 amino acid motifs were determined using Seq2Logo 2.0 [57] with Shannon logo type and without weight on priors.

Suppl Fig.4

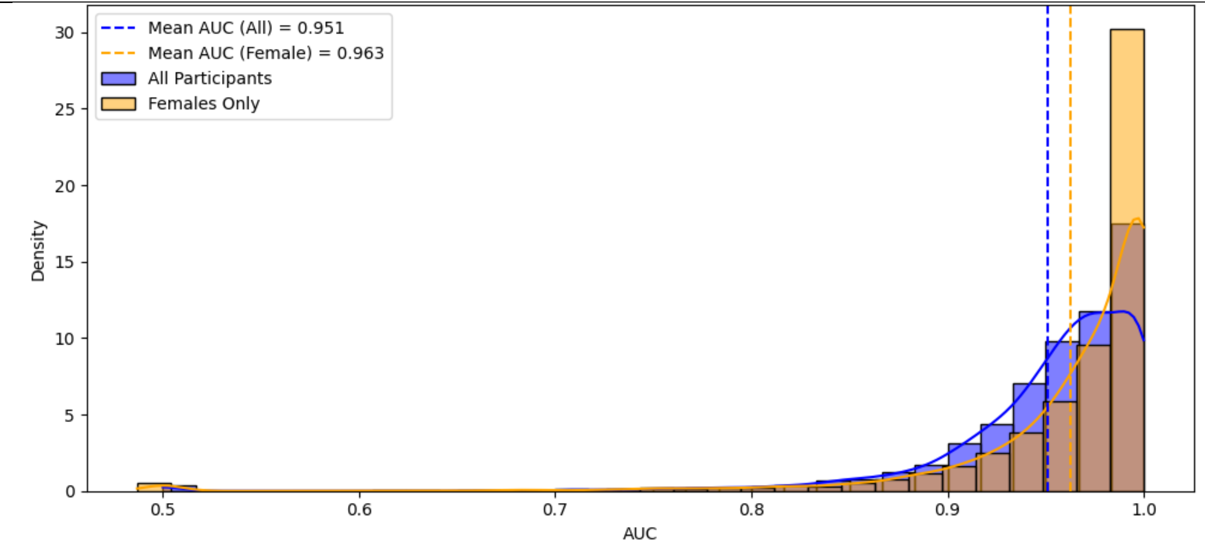

Suppl. Fig. 4 *Bootstrap validation for all-participants and female-only of L1 regression models*

Replacement bootstrapping was performed for both regression models with n=10.000 resampling iterations. For the all-participants model a mean AUC of 0.9513 was calculated with a 95% confidence interval of 0.8097-1.00. For the female-only model a mean AUC of 0.9627 was calculated with a 95% confidence interval of 0.7917, 1.00.

Suppl Fig.5

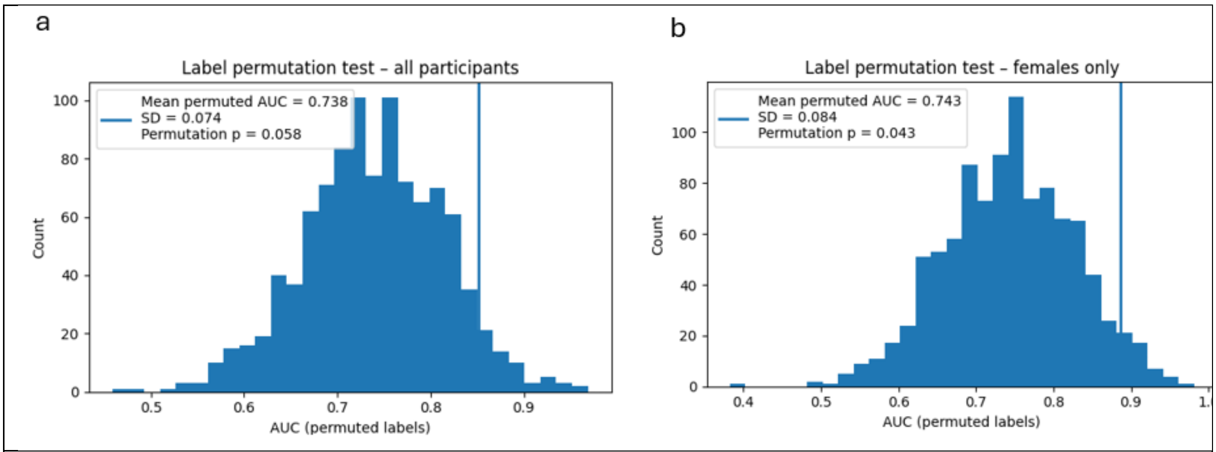

Suppl. Fig. 5 *Label permutation testing of L1-regularized logistic regression models*

Label permutation testing of L1-regularized logistic regression models was performed for the a) both gender regression model and b) the female-only regression model. Outcome labels were randomly permuted 1,000 times and model performance was evaluated . Histograms show the distribution of AUCs obtained under label permutation; the vertical line indicates the observed AUC. The mean and standard deviation of the permuted AUCs and the permutation p-value is indicated.
